# Effects of Biochar Amendment on CO_2_ Emissions from Paddy Fields under Water-Saving Irrigation

**DOI:** 10.3390/ijerph15112580

**Published:** 2018-11-18

**Authors:** Shihong Yang, Zewei Jiang, Xiao Sun, Jie Ding, Junzeng Xu

**Affiliations:** 1 State Key Laboratory of Hydrology-Water Resources and Hydraulic Engineering, Hohai University, Nanjing 210098, China; xjz481@hhu.edu.cn; 2 College of Agricultural Engineering, Hohai University, Nanjing 210098, China; 171302060019@hhu.edu.cn (Z.J.); sx1027@hhu.edu.cn (X.S.); hhudingjie@hhu.edu.cn (J.D.)

**Keywords:** water-saving irrigation, biochar, CO_2_, Q_10_, paddy field

## Abstract

The role of carbon pool of biochar as a method of long-term C sequestration in global warming mitigation is unclear. A two-year field study was conducted to investigate the seasonal variations of CO_2_ emissions from water-saving irrigation paddy fields in response to biochar amendment and irrigation patterns. Three biochar treatments under water-saving irrigation and one biochar treatment under flooding irrigation were studied, and the application rates were 0, 20, 40, and 40 t ha^−1^ and labeled as CI + NB (controlled irrigation and none biochar added), CI + MB (controlled irrigation and medium biochar added), CI + HB (controlled irrigation and high biochar added), and FI + HB (flood irrigation and high biochar added), respectively. Results showed that biochar application at medium rates (20 t ha^−1^) decreased CO_2_ emissions by 1.64–8.83% in rice paddy fields under water-saving irrigation, compared with the non-amendment treatment. However, the CO_2_ emissions from paddy fields increased by 4.39–5.43% in the CI + HB treatment, compared with CI + NB. Furthermore, the mean CO_2_ emissions from paddy fields under water-saving irrigation decreased by 2.22% compared with flood irrigation under the same amount of biochar application (40 t ha^−1^). Biochar amendment increased rice yield and water use efficiency by 9.35–36.30% and 15.1–42.5%, respectively, when combined with water-saving irrigation. The CO_2_ emissions were reduced in the CI + MB treatment, which then increased rice yield. The CO_2_ emissions from paddy fields were positively correlated with temperature. The highest value of the temperature sensitivity coefficient (Q_10_) was derived for the CI + MB treatment. The Q_10_ was higher under water-saving irrigation compared with flooding irrigation.

## 1. Introduction

The global scientific community has generally regarded the greenhouse effect as a major environmental concern. The concentration of CO_2_ in the atmosphere has risen to the highest level in 800,000 years [1]. The agroecosystem plays an important role in the budget, which account for 30%, of anthropogenic greenhouse gas (GHG) emissions [2]. As one of the world’s main land types, paddy fields in monsoonal Asia are also considered significant contributors of GHG to the environment [3]. Moreover, rice planting areas account for approximately 26.4% of the total planting area in China, in which rice is one of the main grain crops. Therefore, further studies are necessary to identify the appropriate regulation of GHG emissions and emission reduction measures.

Most previous studies on paddy fields and CO_2_ emissions have focused on emission regulation, cultivation method, and fertilization. By contrast, few studies have examined the impact of biochar on CO_2_ emissions. For instance, the diurnal patterns of CO_2_ emissions from paddy fields were reported to be unimodal [4]. Previous studies have also shown that adopting less intensive tillage systems can effectively reduce CO_2_ emissions [5]. Some researchers have suggested that no-tillage measures have minimal effect on CO_2_ emissions from rice fields, that is, the CO_2_ emission fluxes markedly decreased after the long-term application of organic fertilizers in lieu of chemical fertilizers while increased with the shift from non-organic fertilizers to organic fertilizers [6].

Heightened global interests—especially in the field of agriculture research—have focused on the biochar, which is derived from biomass by artificial pyrolysis, and its potential to help mitigate global warming, improve crop productivity, and absorb heavy metals [7]. Some studies have found that biochar has the potential to reduce the decomposition of soil organic matter to counteract the release of CO_2_ [8], increase grain yield, and improve water utilization efficiency [9,10]. The chemical stability of biochar has a carbon sequestration effect, which slows down the greenhouse effect and, thus, can be used as one of the CO_2_ emission reduction measures in farmlands [11]. Biochar reportedly has a certain inhibitory effect on CO_2_ emissions from paddy fields, especially in the late growth stage of rice [12]. However, most of these previous studies have concentrated on flooding irrigation paddy fields; thus, further studies are necessary to identify the effects of biochar amendment on CO_2_ emissions in water-saving irrigation paddy fields. Water-saving practices, especially controlled irrigation, have become one of the basic national policies in China that require wide promotion, considering the availability of measures to mitigate GHG emissions while increasing the crop production [13]. Accordingly, a two-year field study was conducted with the following objectives: (1) to estimate the effects of biochar application on CO_2_ emission fluxes and cumulative CO_2_ emissions of rice fields under water-saving irrigation; (2) to discuss the effects of biochar application on rice yield and water use efficiency of paddy fields under water-saving irrigation; and (3) to evaluate the effects of temperature on CO_2_ emissions from paddy fields under water-saving irrigation. If evidence shows that biochar stimulates CO_2_ emissions, maintaining high rice yields under water-saving irrigation, then the climate mitigation potential of this agronomical practice may be even more successful than expected. This study also aims to provide scientific basis for reducing GHG emissions and improve the utilization efficiency of water resources in paddy fields. In addition, the sensitivities of CO_2_ emissions to temperature are to be quantified under different biochar application rates and water management practices.

## 2. Materials and Methods

### 2.1. Experimental Site

The study was conducted in 2016 and 2017 at the Kunshan Irrigation and Drainage Experiment Station (31°15′15″ N, 120°57′43″ E) in Taihu Lake in China. The study area has a subtropical monsoon climate with a mean annual precipitation of 1097.1 mm, an average annual air temperature of 15.5 °C, and a frost-free period of 234 days·year^−1^. The local sunshine duration is 2085.9 h and used as basis for the rotation of rice and wheat planting. The paddy soil is a hydragric anthrosol, which has a heavy loam texture, with bulk density of 1.32g cm^3^ at 0–30 cm and initial pH of 7.4 at 0–18 cm. The organic matter, total kalium (TK), total phosphor (TP), and total nitrogen (TN) were 2.171%, 2.086%, 0.140%, and 0.179%, respectively.

### 2.2. Experimental Design and Irrigation Management

The experiment comprised two irrigation treatments, namely, controlled irrigation (CI) and flooding irrigation (FI). Three rice–straw biochar treatments were concurrently performed, namely, none biomass biochar at 0 t ha^−1^ (NB); medium biomass biochar at 20 t ha^−1^ (MB) and high biomass biochar at 40 t ha^−1^ (HB) under controlled irrigation and only high biomass biochar at 40 t ha^−1^ (HB) under flooding irrigation. The four treatment combinations shown in Table 1 are controlled irrigation and none biochar added treatment (CI + NB), controlled irrigation and medium biochar added treatment (CI + MB), controlled irrigation and high biochar added treatment (CI + HB), and flood irrigation and high biochar added treatment (FI + HB). Each treatment was designed with three replications. Biochar was provided by Zhejiang Biochar Engineering Technology Research Center and spread in the plots manually and incorporated into soil (approximately 20 cm) using the shovel at 1 day prior to transplantation of rice in 2016. The replicates were established in 12 drainage lysimeters with an area of 5 m^2^ (2.5 m × 2 m) in a complete randomized block design for all four treatments. The rice varieties planted in the study area were Japonica Rice Nanjing 46 in 2016 and Suxiang rice in 2017. The rice was transplanted with 13.0 cm × 25.0 cm hill spacing on 30 June 2016 and harvested on 3 November 2016. The rice was transplanted with the same hill spacing on 30 June 2017 and harvested on 31 October 2017. The experiment was conducted by adopting a local farmer fertilizer practice (Table 2). The TN application rates were 272.9 kg ha^−1^ in 2016 and 292.85 kg ha^−1^ in 2017, respectively.

### 2.3. Yield Measurement, Gas Sampling and Field Measurement

Rice yield was estimated by artificial harvesting the plants per unit area of (1 m^2^) each plot. Each unit was selected by random. After threshing by a thresher (5TS-150A; Hangzhou, China), the rice per unit area was collected and weighted, and then the yield was estimated by multiplying the area.

Each static chamber, which was used in the experiment to collect gas samples, contained six rice strains. The chamber made of 5 mm-thick polyvinyl chloride material comprised two parts, a middle part and an upper part, and both parts had heights of 60.0 cm and floor areas of 50.0 cm × 50.0 cm. During the sampling period, a layer of aluminum foil was placed outside the chamber to reduce the temperature change caused by solar radiation. The temperature probe was perforated from the closed top chamber, and the side outlet gas pipelines were placed approximately 30 cm and 150 cm inside and outside the chambers, respectively. A 60 mL plastic syringe with a three-way valve was placed outside the chamber to extract gas samples.

After rice transplanting, the concentrations of CO_2_ gas samples were measured between 10:00 a.m. and 11:00 a.m. in 5-day intervals before September and in seven-day intervals after September. In particular, samples were collected on the 2nd, 4th, 6th, and 8th days after fertilizer application. Before the gas samples were extracted and the syringe was connected to the static chamber, on-site air was used for washing (2–3 times) to eliminate interference. Moreover, the static chamber was covered for nearly 30 min before testing. The researchers also ensured that the static box, syringe, and Tedlar gas sampling bag to be used for storing and transporting the gas samples were fully connected. The concentrations of CO_2_ were analyzed immediately (within 48 h) after gas sampling by using a gas chromatograph (Agilent 7890A; Agilent, Santa Clara, CA, USA) in the laboratory. The cumulative CO_2_ emissions were calculated on the basis of GHG emissions versus sampling days:

(1) $$F=\rho \cdot h\cdot \frac{273}{273+T}\cdot \frac{\mathrm{dC}}{\mathrm{dt}},$$

where F is the CO_2_ emission flux (mg m^−2^ h^−1^); ρ is the CO_2_ gas density at standard state (1.973); h is the effective height of the top static chamber above the soil surface (m, h = 0.41 m); T is the mean air temperature inside the chamber during sampling (°C); and $\frac{\mathrm{dC}}{\mathrm{dt}}$ is the linear change rate of CO_2_ concentration versus time (mg m^−3^ h^−1^).

A previous study suggested that the temperature sensitivity coefficient (Q_10_) value can effectively reflect the sensitivity of CO_2_ emission flux to temperature change. The temperature sensitivities of soil temperature and air temperature are expressed as Q_s10_ and Q_a10_, respectively. The exponential model fitting formula for soil respiration and Q_10_ were calculated as:

F = a e ^b T^, (2)

Q_10_ = e ^10 b^, (3)

where F is the CO_2_ emission flux (mg m^−2^ h^−1^); T is either soil temperature or air temperature; a is the CO_2_ emission flux (mg m^−2^ h^−1^) at 0 °C; and b is the temperature reaction coefficient.

An automatic soil moisture and temperature automatic measuring system called HOBO was set up to automatically monitor the soil moisture and the temperature of the paddy fields. The water layer was recorded at 8:00 a.m. using vertical rulers which were pre-embedded in the field. The amount of irrigation water was calculated based on the difference in water meter before and after irrigation.

Statistical analysis was conducted in SPSS 22.0 (IBM, Armonk, NY, USA) according to the standard procedures of randomized plotting. The statistical significance was calculated on the basis of F tests and least significant differences at the 0.05 probability level.

## 3. Results

### 3.1. Effects of Biochar Application on CO_2_ Emission Fluxes of Rice Fields

**Figure 1:**
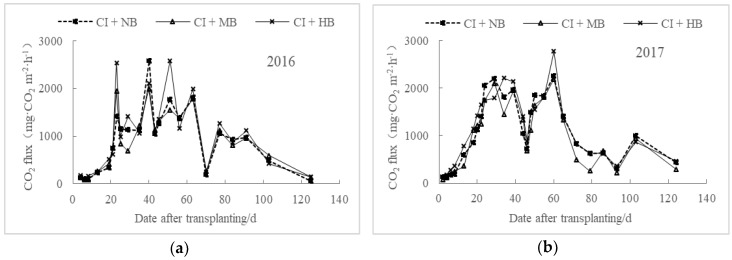
Dynamic changes of CO_2_ emissions from water-saving rice paddies under different biochar application rates in 2016 (**a**) and 2017 (**b**).

Under different biochar applications, the regulation of CO_2_ emissions from paddy fields under water-saving irrigation was generally consistent, and no significant difference was observed between the 2016 and 2017 data (Figure 1). The CO_2_ emission fluxes continued to increase before 24 DAT (date after transplanting) and remained at relatively high levels during 24–60 DAT in 2016 and 24–63 DAT in 2017, and then gradually decreased. Applying moderate amounts of biochar reduced CO_2_ emissions in the paddy fields, whereas high contents of biomass biochar promoted CO_2_ emissions, which may be due to the upper limit of carbon sequestration function of biochar [14]. The average CO_2_ emission fluxes in 2016 were 882.74 mg m^−2^ h^−1^ for CI + MB and 1006.19 mg m^−2^ h^−1^ for CI + HB, which were 2.98% lower and 10.59% higher than those of CI + NB, respectively. Meanwhile, the fluxes in 2017 of CI + MB and CI + HB were 6.62% lower and 9.89% higher than those of CI + NB, respectively. The patterns of CO_2_ emissions have multimodal features. Majority of the CO_2_ emissions were observed in the early growth stage, and the peak fluxes were nearly the same time for different treatments. The first peak of CO_2_ emissions occurred in all three treatments of CI + NB, CI + MB, and CI + HB during the tillering stage. The peak value of CO_2_ emissions of CI + MB and CI + HB in 2016 were 1941.27 mg m^−2^ h^−1^ and 2581.57 mg m^−2^ h^−1^, a decrease by 24.64% and an increase by 0.22% compared with those of CI + NB, respectively. In 2017, the emission peaks of CI + MB and CI + HB were 2185.7 mg m^−2^ h^−1^ and 2771.49 mg m^−2^ h^−1^, which were 2.96% lower and 23.04% higher than those of CI + NB, respectively.

**Figure 2:**
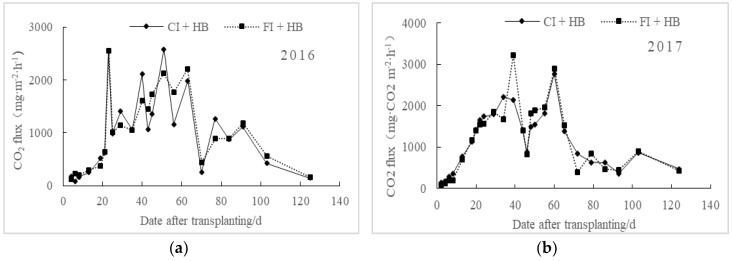
Effects of irrigation treatments on CO_2_ emission fluxes from biochar applied paddy fields in 2016 (**a**) and 2017 (**b**).

No significant difference exists between the regulations of soil CO_2_ emissions under different irrigation treatments, which keep the same upward and downward trend (Figure 2). The CO_2_ emission fluxes of flooding irrigation treatment (FI + HB) were higher than those of controlled irrigation (CI + HB), which may be related to the increase of soil dissolved organic carbon (DOC) content, substrate and microorganism activities, and acceleration of soil mineralization under flooding irrigation. The mean CO_2_ emission fluxes of water-saving irrigation paddies (CI + HB) were 1006.19 mg·m^−2^·h^−1^ in 2016 and 1156.81 mg·m^−2^·h^−1^ in 2017, which were 2.07% and 2.22% lower than those of FI + HB, respectively. These findings may be attributed to different fertilization treatments and climate conditions between the two study years.

### 3.2. Effects of Biochar on Cumulative CO_2_ Emissions from Rice Fields at Different Growth Stages

**Figure 3:**
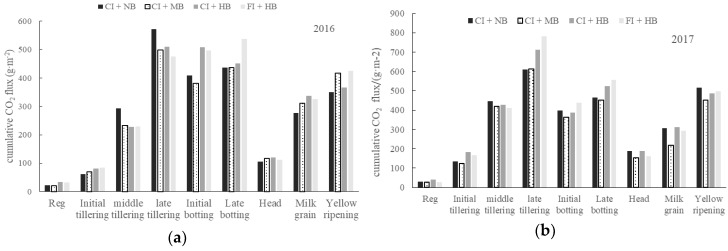
CO_2_ emission fluxes at different growth stages of rice under different amounts of biochar application in 2016 (**a**) and 2017 (**b**) (Reg denotes re-greening stage and Head denotes heading and flowering stage).

The CO_2_ emission fluxes at different growth stages of paddy fields under different biochar applications are shown in Figure 3. No significant difference was observed between 2016 and 2017. Compared with the contrast treatment (CI + NB), the moderate amount of biochar application (CI + MB) inhibited CO_2_ emissions, while the high amount of biochar application (CI + HB) promoted CO_2_ emissions. The general trend can be described as an increase after transplanting, maintained at the high level during the middle tillering stage until the jointing and booting stage, and then a gradual decrease. The CO_2_ emissions from paddy fields were mainly concentrated in the middle tillering stage, late tillering stage, and jointing and booting stage. The main peaks appeared in the late tillering stage, which may be related to the water management in the field. Soil moisture was saturated in the initial and middle stages of tillering. Moreover, rainfall in the heading flowering period was not conducive to CO_2_ emissions. As a result, the CO_2_ emissions were concentrated in the middle-tillering stage until the booting stage. The cumulative CO_2_ emissions of the milk grain stage and the yellow ripening stage were relatively large, and they were mainly related to the long duration of the two phases. The duration of the milk grain stage and the yellow ripening stage reached 19 days and 31 days, respectively.

The cumulative fluxes of CO_2_ of paddy fields were affected by irrigation treatments (Table 3). In 2016, the cumulative CO_2_ emission fluxes during the entire rice growth period were 2484.66 g m^−2^ for CI + MB and 2636.91 g m^−2^ for CI + HB, which were 1.64% lower and 4.39% higher compared with those of CI + NB, respectively, which may be attributed to that flood irrigation increased DOC content, substrate activity, and accelerated soil mineralization. In 2017, the cumulative CO_2_ fluxes were 2820.71 g m^−2^ for CI + MB and 3262.03 g m^−2^ for CI + HB, which were 8.83% lower and 5.43% higher compared with those of CI + NB, respectively. Consequently, the application of medium amounts of biochar has slightly reduced the cumulative fluxes of CO_2_, and the application of high biomass biochar has significantly increased CO_2_ emissions, which are consistent with the results of previous research [15,16].

### 3.3. Effects of Biochar Application on Rice Yield and Water Use Efficiency (WUE)

There was significant increase of the paddy yield after applying biochar under water-saving irrigation. The yields of CI + MB and CI + HB in 2016 were 8070 kg m^−2^ and 8550 kg m^−2^, respectively, which increased by 9.35% and 15.85% respectively compared with CI + NB. The yield of CI + MB and CI + HB in 2017 was 6662 kg m^−2^ and 7321 kg m^−2^, which was 24.03% and 36.30% higher than CI + NB respectively (Table 4). In addition, the application of biochar increased the irrigation water use efficiency of rice. The irrigation water use efficiency of CI + MB and CI + HB in 2016 increased by 15.1% and 19.0%, while increased by 33.4% and 42.5% in 2017, compared with CI + NB respectively. The yield of water-saving irrigation did not drop, even increased in 2017. Meanwhile, water-saving irrigation significantly reduced the amount of irrigation water, which decreased by 55.1% and 40.5% respectively in 2016 and 2017. Thus, the water use efficiency of irrigation in the paddy fields was significantly improved under water-saving irrigation, which was 2.10 and 1.70 times that of flooding irrigation in 2016 and 2017, respectively.

### 3.4. Effects of Temperature on CO_2_ Emissions from Paddy Fields

**Figure 4:**
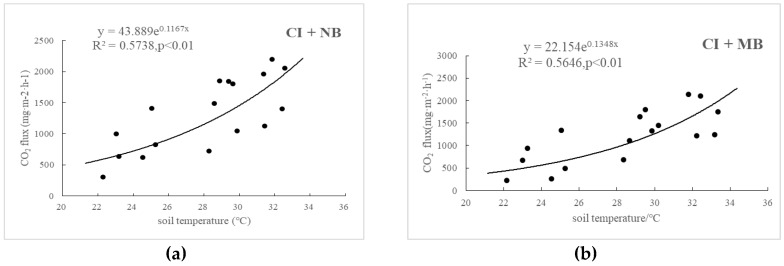

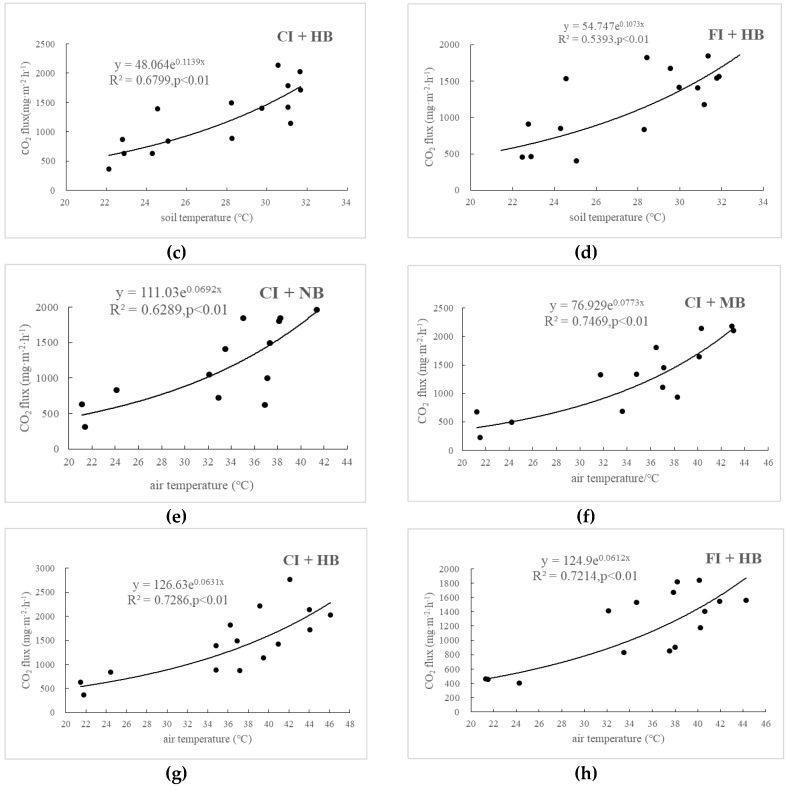
Effects of soil temperature on CO_2_ emissions in CI + NB (**a**), CI + MB (**b**), CI + HB (**c**), FI + HB (**d**) and relationship between CO_2_ emissions and air temperature in CI + NB (**e**), CI + MB (**f**), CI + HB (**g**), and FI + HB (**h**).

Temperature remarkably affected the CO_2_ emissions of paddy fields, and the CO_2_ fluxes of these paddy fields presented significant exponential positive correlations with air temperature and soil temperature (Figure 4). In addition, the correlation coefficient between CO_2_ emissions flux and air temperature was greater than that between CO_2_ emissions flux and soil temperature, a result that is consistent with those of previous studies. These findings may be attributed to the high influence of air temperature on crop respiration. In other words, soil temperature was the main factor that affected CO_2_ emissions of paddy fields. Soil temperature sensitivity (Q_s10_) was greater than that of air temperature sensitivity (Q_a10_), which can be attributed to the air temperature being the influencing factor of soil temperature (Table 5). The average soil temperature of CI + NB, CI + MB and CI + HB (27.79 °C) was higher than that of FI + HB (27.68 °C), which might be attributed to the absence of water protection in controlled irrigation. Compared with that of the control, the application of medium amounts of biochar increased the temperature sensitivity of CO_2_ emissions from water-saving irrigation paddy fields, with the maximum Q_s10_ and Q_a10_ values at 3.85 and 2.17, respectively; however, no significant difference was observed in the case of high biomass. Furthermore, the temperature sensitivity of CO_2_ emission from controlled irrigation of paddy fields was higher than that from flooding irrigation, and the Q_s10_ was 3.12 and the Q_a10_ was 1.88 for of CI + HB, which were 1.07 times and 1.02 times of FI + HB, respectively. The water layer of the paddy field under flooding irrigation played a role of barrier and thermal insulation, and thus, temperature sensitivity was lower.

## 4. Discussion

The results show that medium biomass biochar (CI + MB) inhibits CO_2_ emissions, whereas high biomass biochar (CI +HB) promotes CO_2_ emissions, which are consistent with those of previous research [11]. The alkaline biochar is microbiologically inert and difficult to be used by soil microbes after addition to the soil, which subsequently increases soil pH [17]. The addition of biochar significantly affects CO_2_ absorption resulting from crop respiration, changes in microbial community structures of paddy soil, microbial activity, increase in the ratio of bacterial to fungi [18], and inhibition of CO_2_ emissions. Previous studies have also shown that biochar can be combined with soil aggregates after application to reduce contact area with the external system, reduce reactivity, and contribute to the formation of organic macromolecules that are difficult to use, such as carbohydrates and aromatics, thereby further reducing organic carbon and inhibiting CO_2_ emissions [16]. However, there is an upper limit to the carbon sequestration function of biochar, although its role as a long-term carbon pool may partly counterbalance the increase of carbon emissions caused by the decomposition of organic matter [19]. Therefore, CO_2_ emissions increase under the conditions of high biochar application. In addition, extant studies on the effect of biochar application to the soil on CO_2_ fluxes have not always been consistent, e.g., biochar increases CO_2_ emissions, but this finding has not been reported in all experiments [20,21]. In general, the reduction of CO_2_ emissions from biochar is uncertain [22], and thus, further research is needed to demonstrate the exact relationship.

Differences in CO_2_ fluxes between different water management treatments were investigated with high amounts of biochar application (40 t ha^−1^). Under the condition of high biochar application, the CO_2_ emission flux under water-saving irrigation decreased by approximately 2% compared with that under flooding irrigation. The application of high amounts of biochar under flood irrigation resulted in the promotion of CO_2_ emissions. This finding can be attributed to the increased contents of organic carbon and solute carbon after the application of biochar, which then led to increased soil CO_2_ emissions. The amount of CO_2_ emitted by the soil was nearly equal to the amount of organic and inorganic carbon decomposition during biochar releases [8]. Unlike flooding irrigation, water-saving irrigation reduced CO_2_ emissions from paddy fields, which may be related to soil physicochemical properties and carbon and nitrogen components. Furthermore, the addition of biochar significantly reduced soil mineralization, while soil mineralization was the main method of CO_2_ emission [16,23]. The analysis of paddy fields with different treatments showed that the soil carbon and nitrogen contents of paddy fields under water-saving irrigation were much less than those under flooding irrigation, which were consistent with the findings of previous studies [24,25]. Moreover, the natural drying of paddy fields under flooding irrigation increased the oxygen content and the degree of oxidation, promoted the decomposition of organic matter in paddy fields, increased the content of soil organic carbon, and provided sufficient substrates and soil microorganisms for rice root respiration, which were beneficial to the diffusion of CO_2_ from the soil to the atmosphere. Compared with the alternating wetting-and-drying irrigation regimes under flooding irrigation, the change in soil moisture of paddy fields under water-saving irrigation was not as drastic, thereby resulting in the decrease of CO_2_ emission fluxes.

Temperature was the most important factor that affected CO_2_ emissions from paddy fields, a result that accords with those of previous studies [26], which shows the significant positive correlation between soil CO_2_ emissions and soil temperature and air temperature. The effect can be explained by the phenomena by which air temperature directly affects root respiration and microbial activity, and then affects CO_2_ emissions. Compared with the control treatment (CI + NB), the application of medium amounts of biochar increased the temperature sensitivity of CO_2_ emissions from paddy fields, but the difference was not significant at high levels, which can be explained by the increase of soil moisture after the application of biochar, after which the increased soil moisture within a certain range increases the temperature sensitivity of soil respiration [27,28]. Biochar application also promoted the root growth of rice; accordingly, root respiration was reported to be an important component of soil respiration, and it has high temperature sensitivity [29]. Therefore, the temperature sensitivity of CO_2_ emissions from paddy fields was significantly high under the application of medium amounts of biochar. Meanwhile, high biomass biochar significantly increased the soil carbon and nitrogen ratio. Subsequently, the competition between crops and microbes inhibited the respiration of crops and microorganisms and increased the difficulty of soil microbial utilization of substrates, which then reduced the carbon substrate concentration that can be decomposed. According to the Michaelis–Menten equation, soil respiration is an enzymatic reaction, and the temperature sensitivity of soil respiration is positively correlated with substrate concentration [30]. Thus, the temperature sensitivity of soil respiration decreased after the substrate concentration was reduced, which then can offset the increase of soil respiration sensitivity due to soil moisture increase after biochar application. This phenomenon may explain the minimal difference in temperature sensitivity of CO_2_ emissions in paddy fields with high biomass biochar. Irrigation management approaches also affected the temperature sensitivity of CO_2_ emissions from paddy fields, and the temperature sensitivity of controlled irrigation was higher than that of flooding irrigation. This finding may be attributed to the characteristic of flooding-irrigated paddy fields having a long-term state of water layers, i.e., excessive water can block the pores of soil and reduce permeability, which affect oxygen diffusion and inhibit microorganism and root activities. In addition, some studies found that flooded plants (i.e., located below the water surface) have low maintenance respiration rates [31], and the specific heat capacity of water is high; thus, flooded plants are less sensitive to temperature changes.

## 5. Conclusions

This study investigated the effects of different biochar application rates on CO_2_ emissions, rice yield and water use efficiency in paddy fields under water-saving irrigation. Compared with the control (CI + NB), the application of medium amounts of biochar had a certain inhibitory effect (1.64–8.83%) on the CO_2_ emissions from paddy fields under water-saving irrigation, whereas high biochar amendment increased CO_2_ emissions (4.39–5.43%). Under the biochar application at 40 t ha^−1^, the CO_2_ emissions from water-saving irrigation paddy fields were 2.22% lower than that under flood irrigation. The application of biochar increased the rice yield by 9.35–36.3% and water use efficiency by 15.1–42.5% in paddy fields under water-saving irrigation compared with flooding irrigation. In addition, temperature was the most important factor that affected CO_2_ emissions from paddy fields, and the sensitivity of CO_2_ emissions from paddy fields to soil is higher than that of air. The water-free layer management of water-saving irrigation paddy fields caused the higher sensitivity of CO_2_ emissions to temperature by 1.02–1.07 times than flood irrigation. Biochar amendment at moderate addition rates increased the Q_10_ of CO_2_ emissions from water-saving irrigation paddy fields compared with none added treatment, but the difference was not significant at high addition rates. In conclusion, medium biochar application in paddy fields under water-saving irrigation is a recommended field management mode for paddy fields.

## Figures and Tables

**Table 1:** Biochar application in different treatments (t ha^−1^).

Year	Treatments	Biochar Addition (t ha^−1^)
2016 ^a^	CI + NB	0
	CI + MB	20
	CI + HB	40
	FI + HB	40

Notes: ^a^ There was no biochar application in 2017 because biochar can play a long-term role.

**Table 2:** Date and rate of nitrogen fertilization during the rice-growing season (kg N ha^−1^).

Year	Activity	N
2016	Base fertilizer (29 June) ^a^	72 (CF) ^b^
	Tillering fertilizer (16 July)	97.02 (U)
	Panicle fertilizer (11 August)	103.95 (U)
	Total nitrogen	272.99
2017	Base fertilizer (28 June)	84 (CF + U)
	Tillering fertilizer (14 July)	69.6 (U)
	Panicle fertilizer (9 August)	69.6 (U)
	Total nitrogen	292.85

Notes: ^a^ Dates in brackets refer to the time of fertilizer application; ^b^ CF is compound fertilizer (N, P_2_O_5_, and K_2_O contents are 16%, 12%, and 17% for both 2016 and 2017). U is urea (N content is 46.4%).

**Table 3:** Cumulative CO_2_ emissions under different biochar application rates.

Year	Treatments	Cumulative CO_2_ Emissions (g CO_2_ m^−2^)
2016	CI + NB	2526.12
	CI + MB	2484.66
	CI + HB	2636.91
2017	CI + NB	3093.99
	CI + MB	2820.71
	CI + HB	3263.03

**Table 4:** Rice yield and water use efficiency under different treatments.

Year	Treatments	Yield (kg ha^−1^)	Irrigation Volume	WUE (kg m^−3^)
2016	CI + NB	7380 ± 2.635 ^b^	498.0	1.482
	CI + MB	8070 ± 2.215 ^a^	473.0	1.706
	CI + HB	8550 ± 7.190 ^a^	484.7	1.764
	FI + HB	9060 ± 0.020 ^a^	1079.7	0.842
2017	CI + NB	5371 ± 1.445 ^b^	619.0	0.868
	CI + MB	6662 ± 2.135 ^a^	575.5	1.158
	CI + HB	7321 ± 0.005 ^a^	592.0	1.237
	FI + HB	7250 ± 0.600 ^a^	995.5	0.728

Note: Different letters (such as a, b) within a row indicate significant differences (p < 0.05).

**Table 5:** Soil temperature sensitivity (Q_s10_) and air temperature sensitivity (Q_a10_) with different treatments.

Treatments	Q_s10_	Q_a10_	b_s_	b_a_
CI + NB	3.212	1.998	0.117	0.069
CI + MB	3.849	2.166	0.135	0.077
CI + HB	3.124	1.879	0.114	0.063
FI + HB	2.915	1.844	0.107	0.061

Note: b_s_ is the soil temperature reaction coefficient and b_a_ is the air temperature reaction coefficient.
